# Characterization, Identification and Application of Lactic Acid Bacteria Isolated from Forage Paddy Rice Silage

**DOI:** 10.1371/journal.pone.0121967

**Published:** 2015-03-24

**Authors:** Kuikui Ni, Yanping Wang, Dongxia Li, Yimin Cai, Huili Pang

**Affiliations:** 1 Henan Provincial Key Laboratory of Ion Beam Bio-engineering, Zhengzhou University, Henan, China; 2 Animal Physiology and Nutrition Division, National Institute of Livestock and Grassland Science (NILGS), Ibaraki, Japan; Wuhan University, CHINA

## Abstract

There has been growing interest to develop forage rice as a new feed resource for livestock. This study was to characterize the natural population of lactic acid bacteria (LAB) and select potentially excellent strains for paddy rice silage preparation in China. One hundred and twenty-six strains were isolated and screened from paddy rice silage prepared using a small-scale fermentation system, and ninety-nine of these isolates were considered to be LAB based on their Gram-positive and catalase-negative morphology and the production of most of their metabolic products as lactic acid. These isolates were divided into eight groups (A-H) on the basis of their morphological and biochemical characteristics. The Group A to H strains were identified as *Lactobacillus (L*.*) plantarum* subsp. *plantarum* (species ratio: 8.1%), *L*. *casei* (5.1%), *Leuconostoc (Ln*.*) pseudomesenteroides* (11.1%), *Pediococcus (P*.*) pentosaceus* (24.2%), *Enterococcus (E*.*) mundtii* (12.1%), *Lactococcus (Lc*.*) garvieae* (15.2%), *E*. *faecium* (9.1%) and *Lc*. *lactis* subsp. *lactis* (15.2%) based on sequence analyses of their 16S rRNA and *recA* genes. *P*. *pentosaceus* was the most abundant member of the LAB population in the paddy rice silage. A selected strain, namely *L*. *casei* R 465, was found to be able to grow under low pH conditions and to improve the silage quality with low pH and a relatively high content of lactic acid. This study demonstrated that forage paddy rice silage contains abundant LAB species and its silage can be well preserved by inoculation with LAB, and that strain R 465 can be a potentially excellent inoculant for paddy rice silage.

## Introduction

Paddy rice has been proven to be economically viable not only as a main dish for human consumption but also as a forage crop for animals [1–3]. Southeast Asia and China are the most important rice-producing areas in the world. The rice planting area in China accounts for approximately 20% of the world’s total area and 23% of all cultivated land in China [4]. With an increase in the population of China and improvements in the living standards, the resulting growth in the demand for animal products has resulted in increased utilization of forage crops, such as paddy rice.

Ensilage technology has long been used to preserve forage crops for livestock. Well-preserved silages are dependent on appropriate fermentation after storage, which results in low pH and the production of sufficient acid to inhibit the growth of undesirable microorganisms [5,6]. During the process of silage fermentation, LAB involved in adequate acidification play an important role in the production of higher-quality silage, and different types of LAB exhibit different effects on the silage quality [7]. The natural populations of LAB in fresh crops are often heterofermentative and low in number [8,9]. Thus, homofermentative bacteria can be used to improve silage preservation by accelerating and enhancing the initial phase of the conservation process via the fermentation of water-soluble carbohydrates (WSC) into lactic acid with a subsequent rapid decrease in pH. Recently, to prevent the aerobic deterioration of silage, heterofermentative LAB species, such as *L*. *brevis* and *L*. *buchneri*, have been developed as silage additives [10].

From a microbiological perspective, there is little information available regarding the microbial ecology of the enormous resource of paddy rice silage in China. If identified, LAB inoculants of paddy rice silage can be evaluated for their potential ability to improve silage quality.

Thus, the goals of the present study were to isolate, screen and identify the LAB that colonize paddy rice silage during the process of the fermentation. The isolates were identified biochemically, and selected representative strains were identified using their 16S rDNA sequence and an analysis of the *recA* gene sequence amplification product. In addition, some of the evaluated excellent LAB strains were used to inoculate rice silage to determine their effect on the fermentation quality.

## Materials and Methods

### Isolation and characterization of LAB from paddy rice silage

The whole crop paddy rice (*Oryza sativa* L.) was collected at the fully ripe stage from a farm in Henan Province, China in June, 2012 and 2013. The permission of the sampling location was issued by Rice Research Institute, Xinxiang Academy of Agricultural Science. Silages were prepared using a small scale system, approximately 100g portions of forage material chopped into about 20-mm length and packed into plastic bags (N-9, Asahi Kasei Co., Ltd., Japan). The bags were sealed with Sharp Vacuum Seal/Package (SQ-202, Sharp Co., Ltd., Japan), and the plastic bags were stored in a room at ambient temperature.

Silage samples were collected at days 3, 7, 30 and 60 during the ensiling process. The samples (10g) were blended with 90ml of sterilized water, and serially diluted from 10^-1^ to 10^-5^ in sterilized water. The number of LAB were measured by plate count on lactobacilli de Man, Rogosa, Sharpe (MRS) agar incubated at 30°C for 48 h under anaerobic conditions (DG 250/min MACS; Don Whitley Science; England). One hundred and twenty-six colonies were selected randomly from the plates containing between 30 and 300 colonies, and each LAB colony was isolated and purified twice by streaking on MRS agar plates. Pure cultures were grown on MRS agar at 30°C for 24 h, and then the purified strains were stored at -80°C in nutrient broth (Difco) for further examination.

### Morphological, physiological and biochemical tests of LAB

Morphological, physiological and biochemical tests of LAB morphology and Gram-staining response were examined after 24 h of incubation on MRS agar. Catalase activity and gas production from glucose were determined using the methods of Kozaki *et a*l [11]. Growth at different temperatures was observed in MRS broth after incubation at 5 and 10°C for 14 days, and at 45 and 50°C for 7 days. Growth at pH 3.0, 3.5, 4.0, 4.5 and 7.0 was observed in MRS broth after incubation at 30°C for 7 days. Salt tolerance of LAB was tested in MRS broth containing 3.0 and 6.5% NaCl at 30°C for 2 days. Carbohydrate assimilation and fermentation of 49 compounds with one control were identified on API 50 CH strips (bioMerieux, Tokyo, Japan).

The preliminary identification of the LAB isolates based on the phenotypic characteristics was performed according to the criteria of Bergey′s Manual of Determinative Bacteriology.

### 16S rRNA gene sequencing and *recA* gene PCR amplification

Cells grown at 30°C for 24h in MRS agar were used for 16S rRNA gene sequence. The 16S rRNA gene sequence coding region was amplified by PCR in a PCR thermal cycler. The sequences of the PCR products were determined directly with a sequencing kit using the prokaryotic 16S ribosomal DNA universal primers 27F (5’-AGAGTTTGATCCTGGCTCAG-3’) and 1492R (5’-GGTTACCTTGTTACGACTT-3’) [12]. Sequence similarity searches were performed using the DNA Database of Japan (DDBJ) and the Basic Local Alignment Search Tool (BLAST). The sequence information was then imported into the Clustal X 1.81 software program (Hitachi Software Engineering Co., Japan) for assembly and alignment. The 16S rDNA sequences of R strains were compared with sequences from type LAB strains held in the DDBJ. Nucleotide substitution rates (*K*unc values) were calculated and phylogenetic trees were constructed using the neighbor-joining method. *Bacillus subtilis* NCDO 1769^T^ was used as an outgroup organism. The topologies of trees were evaluated using bootstrap analysis of the sequence data with Molecular Evolutionary Genetic Analysis (MEGA) 4 software, based on 1,000 random resampling [12]. These sequences were aligned with the type published sequences from DDBJ, GenBank and the European Molecular Biology Laboratory (EMBL).

For further discrimination of strains in the *L*. *plantarum* group, a multiplex PCR assay was performed with the *recA* gene-based primers: *para*F (5′-GTCACAGGCATTACGAAAAC-3′), *pent*F (5′-CAGTGGCGCGGTTGATATC-3′), *plan*F (5'-CCGTTTATGCGGAACACCTA-3′), and pREV (5′-TCGGGATTACCAAACATCAC-3′). The PCR mixture and amplifications were performed as described by Torriani *et al* [13].

The nucleotide sequences for the 16S rDNA described in this report were deposited with GenBank under accession nos. AB921218, AB921219, AB921220, AB921221, AB921222, AB921223, AB921224 and AB921225 for the strains R 413, R 419, R 420, R 421, R 422, R 423, R 442 and R 465, respectively.

### Preselection of LAB strains based on metabolite production (Data not shown)

First, the ninety-nine LAB strains were cultivated in MRS broth for 24h at 30°C. The paddy rice broth was crushed out from 5000g of fresh paddy rice mixed with 10000 ml deionized water in a squeezer, then filtered and sterilized (121°C, 15 min). After this period, the inoculum was standardized using a spectrophotometer (600 nm) at an optical density of 1.0. Subsequently, approximately 100 μl of each strain was inoculated into 50 ml of paddy rice broth, which was incubated at 30°C; two replicates were made for each treatment. After 48 h of fermentation, samples of cultures were taken to evaluate metabolite production by HPLC.

### Silage preparation and microbiological analysis

The whole crop paddy rice was collected at the fully ripe stage from a farm in Henan Province, China in September, 2013 and June, 2014. Silages were prepared using a small scale system, approximately 100g portions of forge material chopped into about 20-mm length and packed into plastic bags (N-9, Asahi Kasei Co., Ltd., Japan). Strain R 422 (*L*. *plantarum* subsp. *plantarum*), R 427 (*L*. *plantarum* subsp. *plantarum*) and R 465 (*L*. *casei*) were selected as additives at 1.0×10^6^ colony forming units (cfu)/g of fresh matter (FM) to paddy rice silage. Experimental treatments included: control silage without LAB, the paddy rice + R 422 silage, paddy rice + R 427 silage and paddy rice + R 465 silage. The bags were sealed with Sharp Vacuum Seal/Package (SQ-202, Sharp Co., Ltd., Japan), and the plastic bags were stored in a room at ambient temperature.

Silage samples were collected after 60 d of storage. The samples (10g) were blended with 90ml of sterilized water, and serially diluted from 10^-1^ to 10^-5^ in sterilized water. The LAB analysis method was same as before. Coliform bacteria were counted on blue light broth agar (Nissui-Seiyaku Co., Ltd., Japan), incubated at 30°C for 48 h. Molds and yeasts were counted on potato dextrose agar (Nissui), incubated at 30°C for 24 h, and yeasts were distinguished from molds and other bacteria by colony appearance and the observation of cell morphology. Bacilli and aerobic bacteria were counted on nutrient agar (Nissui), incubated at 30°C for 24 h under aerobic conditions. Colonies were counted as viable numbers of microorganisms in cfu/g of FM. Three repetitions of each sample were analyzed.

### Fermentation quality for paddy rice silage

After 60d of ensiling, three bags per treatment were opened for analyzing fermentation quality. Dry matter (DM) was analyzed according to AOAC Methods 934.01 [14]. Wet silage (10g) was homogenized with 90ml sterilized distilled water. The pH was measured with a glass electrode pH meter (pH 213; HANNA; Italy). The ammonia-N was determined by steam distillation of the filtrates. The organic acid contents were measured by high performance liquid chromatography (HPLC, 1200series; Agilent; American).

### Statistical analyses

The variance analysis and multiple comparisons of data were performed by the GLM procedures of SAS (SAS Institute Inc., Cary, NC, US).

## Results

The morphological and physiological properties of representative strains isolated from paddy rice silage

Ninety-nine of the presumptive LAB strains were characterized using phenotypic characteristics (Table 1) and sugar fermentation assays with API 50 CH strips (Table 2). Among them, 27 (R 381-R 407), 21 (R 408–428), 21 (R 429–449) and 30 (R 450–479) LAB strains were isolated at the 3d, 7d, 30d and 60d of ensiling, respectively. This analysis resulted in the delineation of eight groups of isolates. All of the isolates were Gram-positive and catalase-negative bacteria, unable to grow at 50°C and able to grow at pH 4.0 to 7.0 (Table 1). The Group A and B strains did not produce gas from glucose and could grow at pH 3.5 to 7.0. All the isolated LAB strains were unable to grow at 5.0 and 50.0°C or pH 3.0, and the Groups C and F strains were heterofermentative. However, the other strains showed homofermentative products. The strains in Groups C and D were found to be capable of growing at 10°C, whereas the strains in Groups D, E, and G presented the ability to grow at 45°C. The Group C and F strains were tolerant to low pH 3.5.

**Table 1:** Phenotypic characteristics of representive strains isolated from paddy rice silages.

Characteristics	Group A	Group B	Group C	Group D	Group E	Group F	Group G	Group H
	R 422	R 465	R 413	R 419	R 420	R 421	R 423	R 442
No. of isolates	8	5	11	24	12	15	9	15
Shape	Rod	Rod	Cocci	Cocci	Cocci	Cocci	Cocci	Cocci
Gram stain	+	+	+	+	+	+	+	+
Catalase	-	-	-	-	-	-	-	-
Gas from glucose	-	-	+	-	-	+	-	-
Fermentation type	Homo	Homo	Hetero	Homo	Homo	Hetero	Homo	Homo
Growth at temperature (°C):								
5	-	-	-	-	-	-	-	-
10	+	+	+	+	-	-	w	w
45	-	-	-	+	+	w	+	-
50	-	-	-	-	-	-	-	-
Growth in NaCl:								
3.0%	+	+	+	+	+	w	+	+
6.5%	+	+	-	-	-	-	-	w
Growth at pH:								
3.0	-	-	-	-	-	-	-	-
3.5	+	+	+	w	w	+	-	-
4.0	+	+	+	+	+	+	w	+
4.5	+	+	+	+	+	+	+	+
7.0	+	+	+	+	+	+	+	+

^+^, positive;

^w^, weakly positive;

^-^, negative.

Abbreviations: Homo, homofermentative; Hetero, heterofermentative.

**Table 2:** API 50 CH fermentation patterns of isolated latic acid bacteria from paddy rice silages.

Item	Group A	Group B	Group C	Group D	Group E	Group F	Group G	Group H
	R 422	R 465	R 413	R 419	R420	R 421	R 423	R 442
Glycerol	-	-	-	-	-	-	-	-
Erythritol	-	-	-	-	-	-	-	-
D-Arabinose	-	-	-	-	-	-	-	-
L-Arabinose	+	+	+	+	w	+	+	+
Ribose	+	+	+	+	w	+	-	+
D-Xylose	-	+	+	w	w	+	+	-
L-Xylose	-	-	-	-	-	-	-	-
Adonitol	-	-	-	-	-	-	-	-
*β*-Methyl-xyloside	-	-	-	-	-	-	-	-
Galactose	+	+	+	+	-	+	+	+
D-Glucose	+	+	+	+	-	+	+	+
D-Fructose	+	+	+	+	-	-	-	-
D-Mannose	+	+	+	+	-	+	+	+
L-Sorbose	-	-	-	w	-	-	-	-
Rhamnose	-	-	-	w	-	-	-	-
Dulcitol	-	-	-	-	-	-	-	-
Inositol	-	-	-	-	-	-	-	-
Mannitol	+	-	-	-	-	-	-	-
Sorbitol	+	-	-	-	-	-	-	-
α-Methyl-D-mannoside	+	-	-	-	-	-	-	-
α-Methyl-D-glucoside	-	+	+	-	-	-	-	-
*N*-acetyl glucosamine	+	w	w	-	-	-	-	-
Amygdaline	+	+	-	-	w	+	+	+
Arbutine	+	w	w	+	w	+	+	+
Esculine	+	-	-	+	w	-	-	-
Salicine	+	w	w	+	w	-	-	-
Cellobiose	+	+	+	+	+	-	-	-
Maltose	+	+	+	+	-	-	-	-
Lactose	+	w	w	-	-	-	-	-
Melibiose	+	+	+	-	-	-	-	-
Saccharose	+	+	+	-	-	w	-	w
Trehalose	+	+	+	-	-	+	+	+
Inuline	-	-	-	-	-	-	-	-
Melezitose	+	+	-	+	-	-	-	-
D-Raffinose	w	+	+	w	-	-	-	-
Starch	-	-	-	-	-	-	-	-
Glycogene	-	-	-	-	-	-	-	-
Xylitol	-	-	-	-	-	-	-	-
*β*-Gentiobiose	+	w	w	w	w	-	-	-
D-Turanose	+	+	+	-	-	-	-	+
D-Lyxose	-	-	-	-	-	-	-	-
D-Tagatose	-	-	-	-	-	-	-	-
D-Fucose	-	-	-	-	-	-	-	-
L-Fucose	-	-	-	-	-	-	-	-
D-Arabitol	w	-	-	-	-	-	-	-
L-Arabitol	-	-	-	-	-	-	-	-
Gluconate	+	-	-	-	-	w	w	w
2-ceto-gluconate	-	-	-	-	-	-	-	-
5-ceto-gluconate	-	-	-	-	-	-	-	-

^+^, positive;

^w^, weakly positive;

^-^, negative.

### 16S rRNA gene sequence analysis

**Fig 1:**
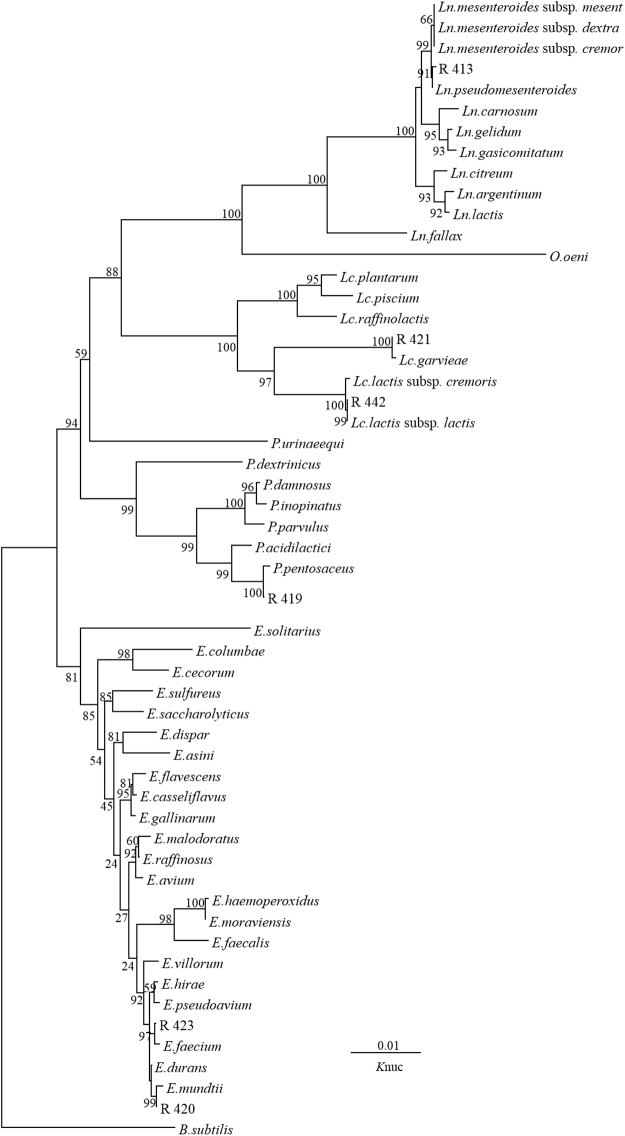
Phylogenetic tree showing the relative positions of isolates R 413, R 419, R420, R 421, R 423 and R 442 as referred by the neighbor-joining method of complete 16S rDNA sequences. Bootstrap values for 100 replicates are shown at the nodes of the tree. *Bacillus subtilis* is used as an outgroup. The bar indicates 1% sequence divergence. *Ln*. = *Leuconostoc*; *Lc*. = *Lactococcus*; *P*. = *Pediococcus*; and *E*. = *Enterococcus*. *K*nuc = nucleotide substitution rate.

**Fig 2:**
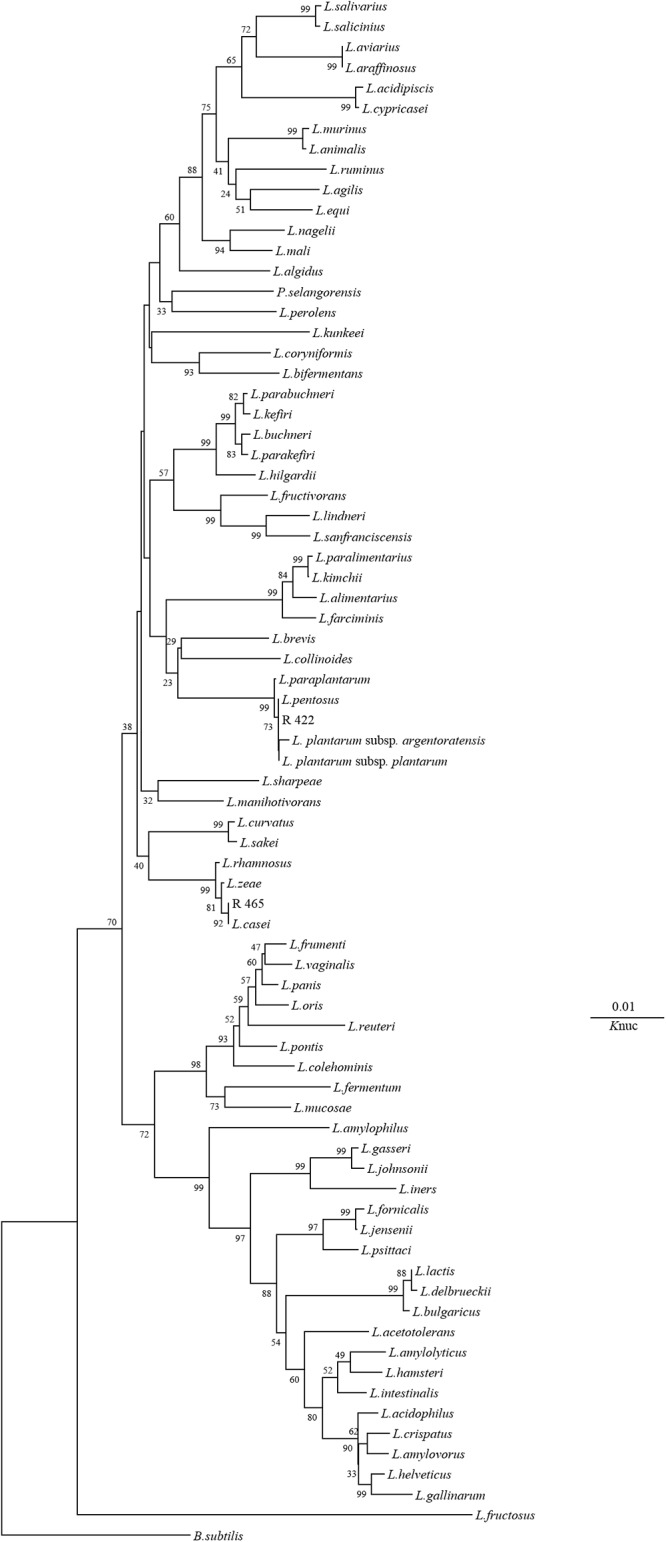
Phylogenetic tree showing the relative positions of isolates R 422 and R 465 as referred by the neighbor-joining method of complete 16S rDNA sequences. Bootstrap values for 100 replicates are shown at the nodes of the tree. *Bacillus subtilis* is used as an outgroup. The bar indicates 1% sequence divergence. *L*. = *Lactobacillus*. *K*nuc = nucleotide substitution rate.

As shown in Figs. 1 and 2, according to the phylogenetic analysis, a representative strain in Group A, namely R 422, clearly belonged to the genus *Lactobacillus* because it was grouped in the *L*. *plantarum* cluster, which included *L*. *pentosus*, *L*. *plantarum* subsp. *plantarum*, *L*. *plantarum* subsp. *argentotatensis* and *L*. *paraplantarum*, on the phylogenetic tree (Fig. 2). The Group B strain R 465 was categorized in the *L*. *casei* cluster. The Group C strain R 413 was closely related to the *Ln*. *pseudomesenteroides* species. The Group D strain R 419 was categorized in the *Pediococcus* cluster because it was grouped with *P*. *pentosaceus* on the phylogenetic tree, and this grouping was supported with a bootstrap value of 100%. Strain R 420 in Group E and strain R 423 in Group G were categorized into the *Enterococcus* cluster with *E*. *mundtii* and *E*. *faecium*. Strain R421 in Group F can be assigned to *Lc*. *garvieae*, and this finding was supported with a bootstrap value of 100%. In Group H, *Lc*. *lactis* subsp. *lactis* was the most closely related species.

### Amplification products obtained from the *recA* gene multiplex assay

**Fig 3:**
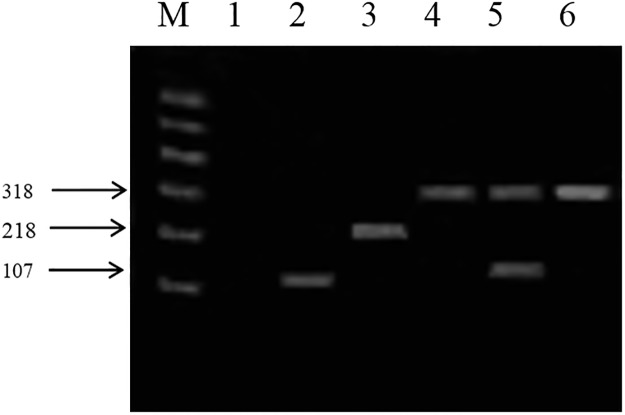
Amplification products obtained from the *recA* multiplex assay. Lane M contained a 600 bp PLUS DNA ladder (Tiangen Biotech Co, Ltd., Beijing, China). Lanes 1, 2, 3, 4 and 5, PCR amplication products from *L*. *casei* JCM 16167^T^ (negative control), *L*. *paraplantarum* JCM 12533^T^, *L*. *pentosus* JCM 1558^T^, *L*. *plantarum* subsp. *plantarum* JCM 1149^T^ and *L*. *plantarum* subsp. *argentoratensis* JCM 16169^T^, respectively; Lane 6, PCR amplification product from R 422.

As shown in Fig. 3, the amplification products shown in lanes 1, 2, 3, 4 and 5 are from *L*. *casei* JCM 16167^T^ (negative control), *L*. *paraplantarum* JCM 12533^T^, *L*. *pentosus* JCM 1558^T^, *L*. *plantarum* subsp. *plantarum* JCM 1149^T^ and *L*. *plantarum* subsp. *argentoratensis* JCM 16169^T^, respectively, and Lane 6 shows the PCR amplification product from strain R 422. Thus, the representative strain R 422 of Group A was clearly identified as *L*. *plantarum* subsp. *plantarum*.

### Fermentation quality of paddy rice silage after 60 d of ensiling in 2013

The fermentation quality of rice silage after 60 d of ensiling are shown in Table 3. The analysis of the chemical composition showed that the contents of DM in the R 422- and R 465-treated silages exhibited a significant (*P* < 0.05) difference compared with the other treatments. The comparison of the fermentation quality with respect to the control demonstrated that the LAB-treated silages presented lower (*P* < 0.05) pH values and a higher (*P* < 0.05) content of lactic acid; in addition, among the four groups, the R 465-treated silages had the lowest pH values and the highest content of lactic acid, and propionic and butyric acids were not detected in any of the treated silages.

**Table 3:** The fermentation quality and viable microorganism of paddy rice silages at 60d of ensiling in 2013.

Item	Silages			
	Control	R 422	R 427	R 465
DM	31.47^B^	33.35^A^	31.24^B^	33.78^A^
pH	6.05^A^	4.34^B^	4.52^B^	3.89^C^
Lactic acid (%FM)	0.33^C^	1.22^B^	1.14^B^	1.70^A^
Acetic acid (%FM)	0.29^B^	0.32^B^	0.56^A^	0.22^B^
Propionic acid (%FM)	ND	ND	ND	ND
Butyric acid (%FM)	ND	ND	ND	ND
LAB (log cfu/g of FM)	6.2^B^	7.6^A^	7.5^A^	7.5^A^
Aerobic bacteria (log cfu/g of FM)	5.2	5.5	5.1	4.5
Colifrom bacteria (log cfu/g of FM)	ND	ND	ND	ND
Yeasts (log cfu/g of FM)	5.1^A^	4.8^A^	5.2^A^	4.1^B^
Molds (log cfu/g of FM)	ND	ND	ND	ND

^A, B, C^Means in the same row with different superscripts in lowercase letter are statistically significantly different (*P* < 0.05).

ND, not detected; FM, fresh matter; LAB, lactic acid bacteria.

### Fermentation quality of paddy rice silage after 60 d of ensiling in 2014

As shown in Table 4, after 60 d of storage, all LAB-inoculated samples showed significantly lower pH values than no-additive groups, reflecting higher lactic acid content (*P* < 0.05). In addition, the inoculated samples showed higher DM recovery. The numbers of LAB in LAB-treated samples showed significantly higher than the control (*P* < 0.05). Using LAB inoculants, the survival of coliform bacteria had dropped to below detectable levels, and R 422 and R 465-treated silages had lower number of yeasts than the others.

**Table 4:** The fermentation quality and viable microorganism of paddy rice silages at 60d of ensiling in 2014.

Item	Silages			
	Control	R 422	R 427	R 465
DM	36.71^B^	39.02^A^	38.89^A^	38.34^A^
pH	6.11^A^	4.25^B^	4.36^B^	3.99^C^
Lactic acid (%FM)	0.42^C^	1.18^B^	1.20^B^	1.83^A^
Acetic acid (%FM)	0.29^B^	0.46^B^	0.36^A^	0.55^B^
Propionic acid (%FM)	ND	ND	ND	ND
Butyric acid (%FM)	ND	ND	ND	ND
LAB (log cfu/g of FM)	7.5^B^	8.7^A^	8.5^A^	8.1^A^
Aerobic bacteria (log cfu/g of FM)	5.2	5.5	5.1	4.5
Colifrom bacteria (log cfu/g of FM)	4.8^A^	ND^B^	ND^B^	ND^B^
Yeasts (log cfu/g of FM)	5.5^A^	4.2^B^	5.1^A^	4.5^B^
Molds (log cfu/g of FM)	ND	ND	ND	ND

^A, B, C^Means in the same row with different superscripts in lowercase letter are statistically significantly different (*P* < 0.05).

ND, not detected; FM, fresh matter; LAB, lactic acid bacteria.

## Discussion

The isolation of bacteria using MRS medium under anaerobic conditions allowed the identification of different morphotypes each time that the silos were opened [15]. In the present study, one hundred and twenty-six strains were screened, and ninety-nine of these isolates were considered LAB as determined by their culture on MRS agar, Gram stain appearance, catalase test results, and lactic acid production from glucose. All of the presumptive LAB were further characterized using sugar fermentation assays with API 50 CH strips. This assay resulted in the delineation of eight groups of isolates, each of which displayed a distinct carbohydrate fermentation pattern. However, the phenotypic procedure used to assign isolates to known species is challenging because it can be difficult to differentiate between species. To identify LAB isolates at the species level, a molecular analysis was performed, and phylogenetic trees were constructed based on the evolutionary distances of their 16S rDNA sequences using the neighbour-joining method. The representative strains of Group B (R 465), Group C (R 413), Group D (R 419), Group E (R 420), Group F (R 421), Group G (R 423) and Group H (R 442) were identified as *L*. *casei*, *Ln*. *pseudomesenteroides*, *P*. *pentosaceus*, *E*. *mundtii*, *Lc*. *garvieae*, *E*. *faecium* and *Lc*. *lactis* subsp. *lactis*, respectively.

Group A strains (representative strain: R 422) were categorized on the phylogenetic tree with *L*. *pentosus*, *L*. *plantarum* subsp. *plantarum*, *L*. *plantarum* subsp. *argentoratensis* and *L*. *paraplantarum*. However, the 16S rDNA sequence analysis was not sufficient to differentiate subspecies in the *L*. *plantarum* group. Thus, other phylogenetic analysis methods were required, such as an analysis of the *recA* gene, which is more variable and has been proposed as a phylogenetic marker for distantly related species [12]. The *recA* gene-specific multiplex PCR analysis revealed that the PCR products of the tested strains were similar to those obtained with *L*. *plantarum* subsp. *plantarum* JCM 1149^T^, indicating that the Group A strains are *L*. *plantarum* subsp. *plantarum*.

The biochemical and phylogenetic analyses revealed that most of the characterized LAB belong to the genera *Lactobacillus*, *Lactococcus*, *Pediococcus*, *Enterococcus* and *Leuconostoc*. The species diversity was observed because eight species were also identified: *P*. *pentosaceus* (24.2%), *Lc*. *garvieae* (15.2%), *Lc*. *lactis* subsp. *lactis* (15.2%), *E*. *mundtii* (12.1%), *L*. *plantarum* subsp. *plantarum* (8.1%), *Ln*. *pseudomesenteroides* (11.1%), *E*. *faecium* (9.1%) and *L*. *casei* (5.1%). However, *Lactobacillus* only accounted for 13.2% of the total LAB microflora. The LAB species identified in this study were common inhabitants of a variety of forage crops and silages. This finding was consistent with our previous investigations and the results of other studies, which showed that the natural fermentation processes in forage crop and grass silages are dominated by *Leuconostoc*, *Lactococcus*, *Enterococcus*, *Pediococcus* and *Lactobacillus* species [16–18]. However, Ennahar *et al* [1] reported that the LAB species of paddy rice silage in Japan also include *P*. *acidilactici* and *Weissella kimchi*, which were not observed in the present study. A plausible reason may be that the bacterial colonization of fresh crops and plants is controlled by many factors, such as different climates [19]. Thus, we may need to collect more paddy rice samples from various locations to build a more global picture of LAB and to determine what inoculant characteristics may be the most important.

Spontaneous fermentation by LAB in silage is a complex microbial process in which the composition of the dominant microbiota changes at different stages, particularly during the first week of ensiling when major changes in the fermentation products and pH occur [20]. In case of missing species that reflect the silage quality, we sampled rice silage after 3 d, 7 d, 30 d and 60 d of ensiling for a period of two years. From this investigation, eight LAB species were found to be present in rice silages, and most of these species belong to cocci. Among epiphytic LAB, cocci (e.g., enterococci, leuconostocs, weissella, pediococci and lactococci) are known to initiate lactic acid production during the early stage of fermentation. During this process, some heterofermentative cocci produce gas from glucose and create aerobic conditions that are suitable for the development of lactobacilli. In contrast to cocci, lactobacilli are important promoters of lactic acid fermentation for a longer time, particularly during the latter stage of ensiling [21,22]. Nevertheless, the lactobacilli identified in this study were found at a markedly lower level in the natural fermentation of paddy rice silage. In this regard, the present results suggest that it is necessary to inoculate paddy rice silage with excellent LAB, particularly lactobacilli, to increase the initial load of the inoculated LAB.

Based on the first step of the screening process (data not shown), two strains (R 422 and R 427) from group A and one strain (R 465) from group B producing the highest amounts of lactic acid in the paddy rice broth were selected as the inoculants for rice silage. Lactic acid is usually the main reason for low pH in high quality silage. Many procedures have been applied to obtain excellent LAB inoculants [23]. In general, researchers will select LAB strains that possess high abilities to survive in various fermentation environment and produce lactic acid as the main product of carbohydrate fermentation, as well as the competiveness between inoculants and natural flora. In this study, the selection was performed at the strain and not the species level. Avila *et al*. [10] evaluated the effect of different LAB species on silage quality and observed that the effect of the inoculant is more related to the strain than the species, and Tohno *et al*. [3] found that the fermentation quality of rice silage inoculated with different LAB strains belonging to the same species differs markedly. Taken together, these two studies support the hypothesis that suitable LAB inoculants should be selected on a strain basis.

The addition of inoculants to silage is an effective way to improve silage quality [24,25]. The function of inoculants is to promote the rapid and efficient utilization of WSC, which results in the intensive production of lactic acid and a rapid decrease in pH and thereby leads to the suppression of non-beneficial microbes [26]. In addition, some studies have found an apparent linkage between silage treated with LAB and animal performance. For example, Cao *et al*. [5] found that vegetable residue silage inoculated with *L*. *plantarum* had the highest *in vitro* DM digestibility and lowest methane production. In this study, after 60 days of storage, the three inoculated strains exerted different effects on the fermentation quality of the paddy rice silages. The silages inoculated with R 465 (*L*. *casei*) had relatively higher contents of lactic acid and lower pH values compared with the control and other treated silages. The lactic acid content and acetic acid content in the uninoculated samples were higher in the current study compared with the results reported by Tohno *et al* [3]. The silages inoculated with R 422 and R 465 presented higher DM contents than the control (*P* < 0.05) in 2013 and 2014. DM is the material remaining after the removal of water and contains the main nutrients found in feeds for animal health. DM loss during the fermentation constitutes the main problems in the ensilage of the forage and the majority of these losses were most likely due to the fermentation process with remainder due to plant and microbial respiration. Cai *et al*. [27] found that inoculation homofermentative LAB may decrease the amount of gas production and DM loss by inhibiting the clostridia and aerobic bacteria, and Carvalho *et al*. [28] found inoculated LAB could inhibit the growth of yeasts which are also responsible for DM losses. In this study, the higher DM losses in the control paddy rice silage were due to higher activity of yeasts (Table 3 and 4), which makes sense because yeasts are known to grow at lower pH values compared with bacteria [29]. In conclusion, this study revealed that forage paddy rice silage contains abundant LAB species and that its silage could be well preserved through inoculation with LAB, which results in a good feed source for livestock diets. *L*. *casei* R 465 originating from rice silage, which showed the highest content of lactic acid and lowest pH value among the three inoculated LAB stains, is considered the most suitable strain for improving paddy rice silage quality and thus is a potential inoculant for the production of silage.
